# A Supercritical Fluid Chromatography/Tandem Mass Spectrometry Method for the Simultaneous Quantification of Metformin and Gliclazide in Human Plasma

**DOI:** 10.4103/0250-474X.62231

**Published:** 2010

**Authors:** Y. K. Agrawal, P. J. Gogoi, K. Manna, H. G. Bhatt, V. K. Jain

**Affiliations:** Institute of Research & Development, Gujarat Forensic Sciences University, Gandhinagar-382 007, India; 1 Institute of Pharmacy, Nirma University, S. G. Highway, Ahmedabad-382 481, India; 2 Chemistry Department, School of Science, Gujarat University, Ahmedabad-380 009, India

**Keywords:** Gliclazide, Metformin, pharmacokinetics, phenformin, SFC/MS/MS

## Abstract

Present study reports the development and validation of a simultaneous estimation of metformin and gliclazide in human plasma using supercritical fluid chromatography followed by tandem mass spectrometry. Acetonitrile:water (80:20) mixture was used as a mobile phase along with liquid CO_2_ in supercritical fluid chromatography and phenformin as an internal standard. The modified plasma samples were analyzed by electro-spray ionization method in selective reaction monitoring mode in tandem mass spectrometry. Supercritical fluid chromatographic separation was performed using nucleosil C_18_ containing column as a stationary phase. The separated products were identified by characteristic peaks and specific fragments peaks in tandem mass spectrometry as m/z 130 to 86 for metformin, m/z 324 to 110 for gliclazide and m/z 206 to 105 for phenformin. The present method was found linear in the concentration ranges of 6.0-3550 ng/ml and 7.5-7500 ng/ml for metformin and gliclazide, respectively. Pharmacokinetic study was performed after an oral administration of dispersible tablets containing 500 mg of metformin and 80 mg of gliclazide using same techniques.

Diabetes mellitus is a very common metabolic disease that is caused by absolute or relative insulin deficiency. Metformin (MET) and gliclazide (GLI) are the most commonly prescribed antidiabetic drugs for type-2 diabetes (noninsulin-dependent diabetes mellitus, NIDDM) in India. Single antidiabetic agent is not sufficient to achieve the normal concentration of insulin level in the blood in hyperglycemic condition. The oral combined dosage form of MET and GLI is best to achieve the adequate control of glucose in the blood[1]. The antidiabetic effects of MET and GLI suggested that, the drugs basically increase the peripheral insulin concentration through stimulation of beta cells in pancreas. Combination of MET and GLI is beneficial in terms of its convenience to patient compliances. Scientific evidences were proved the efficacy of MET and GLI, through the monitoring of plasma drugs concentration during the antidiabetic therapy in diabetic patients[2-4].

Several methods are available for the plasma drugs concentration, optimization of dose and pharmacokinetic study of single and combined dosage forms of MET and GLI; none of the methods are suitable for routine analysis of combined dosage form of MET and GLI. Most of reported methods were less sensitive, complex, tedious, time consuming and extraction or derivatization reactions are required for the analysis[5–7]. Recently, few sensitive methods were reported for individual estimation of MET or GLI using liquid chromatography with mass spectrometry (LC/MS)[8–16]. Some of the methods were reported for the combined dosage form of MET and GLI (500 and 80 mg/ml) also. But, none of the method is available for separation and simultaneous estimations of MET and GLI using SFC/MS/MS method. Supercritical Fluid Chromatography (SFC) method was adopted for the separation of drugs from human plasma. A SFC method has several advantages like rapid separation without using hazardous organic solvents, comparing with conventional chromatographic techniques like GC, HPLC, and LC. The diffusion rate of solutes is very high (ten times greater than simple organic solvents) in supercritical fluid (liquid CO_2_). As a results decrease in resistance to mass transfer in the column and allow to very fast separation. The present work was attempted an isocratic separation and simultaneous estimation of MET and GLI using supercritical CO_2_ doped with acetonitrile: water (80:20) and MS/MS quantification. First time SFC/MS/MS techniques was adopted for the determination, simultaneous estimations and pharmacokinetic study of MET and GLI in human plasma.

## MATERIALS AND METHODS

Metformin, gliclazide and phenformin were procured from generics, Hertz, UK of 99.9% purity. The purity of drugs was reestablished by HPTLC, FTIR and NMR data, which was matched with standard supplied data. Acetonitrile (HPLC grade) and other chemicals were used from E. Merck India. Double distilled water was made in house. All the solvents including double distilled water were passes through the 0.2 micron Millipore membrane and degassing by ultrasonic bath for 1h before use. Carbon dioxide gas (SFC-grade) was supplied by Bombay Carbon dioxide Co, Ahmedabad, India.

### Supercritical fluid chromatography:

**Fig. 1:**
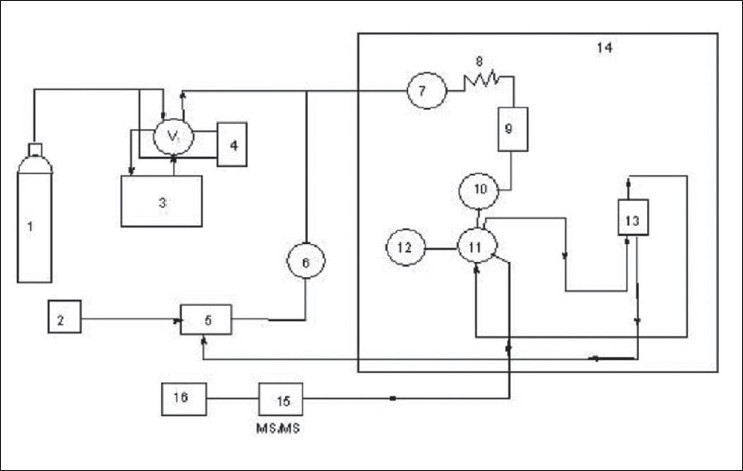
Flow chart of super fluid chromatography/tandem mass spectrometry 1. Liquid carbon dioxide cylinder, 2. Modifier, 3. Cooling circulator, 4. CO_2_ pump with cooling jacket, 5. Modifier pump, 6. Stop valve, 7. Stop valve, 8. Pre heating coil, 9. Mixture, 10. Ejector, 11. Line switching valve, 12. Stop valve, 13. Column, 14. Oven, 15. Detector (MS/MS), 16. back pressure regulator.

A Jasco-900 series (Japan Spectroscopic Co. Ltd., Hachioji, Tokyo, Japan) of supercritical fluid chromatography was employed for the present study. It was equipped with two pumps (PU-980), which were capable to adjust the flow rate (0.001 to 10 ml /min.) of both liquid CO_2_ and modifier. An external loop (model 7125) was equipped with rheodyne injector able to inject 20 μl of liquid sample into the analytical column. The temperature of the column was thermostatically controlled in a column oven (Jasco-Co-965), while inbuilt with a cooling circulator. The outlet pressure was adjusted by back pressure regulator (Jasco-880-81). The working flow chart of SFC/MS/MS is shown in fig. 1.

### The conditions of SFC:

The flow rate and pressure of supercritical CO_2_ were optimized to achieve a good quantitative separation and symmetric shape peaks. The optimized parameters of present investigation were made 20 MPa pressure, 2.0 ml/min flow rate of supercritical CO_2_ and 0.2 ml/min flow rate of modifier. The temperature was maintained at 20 to 60° for better separation. An effective change in retention time and selectivity found at 50°. It also observed that, the change of back pressure and change in density of modifier were directly effect on retention time, while increase in back pressure cause early elution of the analytes. The retention times were observed, 1.8 min. for MET, 2.3 min. for GLI and 2.6 min I.S. Out of the several C_18_ columns, the nucleosil C_18_ was found optimum for quantitative separation of MET and GLI.

#### Mass spectrometry:

API 4000™ system mass spectrometer (Applied Biosystems Sciex, Ontario, Canada) was used in present investigation. It was equipped with exclusive TurboV™ source contained TurboIonSpray^®^ probes which provide advanced linear ion trap technology for highest level of sensitivity. The TurboV ion chamber has embedded ceramic heater technology with improved gas dynamics. Data acquisition and integration were done by Windows-based analyst software.

#### SFC/MS/MS conditions:

The chromatographic separation was under isocratic conditions with a flow rate of 2.0 ml/min. The column effluent was approximately 0.5 ml/min (flow rates as high as 2 ml/min) which was allowed to enter in to the ion chamber on API 4000™ series mass spectrometer. The increased ions yield in to the gas phase given greater sensitivity. The dual ceramic heaters in the ion chamber provide greater efficiency in ionization over the wide range of liquid flow rates from SFC. The atmospheric pressure chemical ionization and Turbo Ion Spray mode were set on for positive and negative ionizations of drugs in 5 KV voltages. The nitrogen curtain gas was supplied and adjusted to a constant flow into the true triple quadrupole. Gas 1 (translates gas) and Gas 2 (nitrogen curtain gas) were set to 30 and 40 PSI units respectively and the source temperature (at set point) was 300°, maximum sensitivity and resolution of the mass spectrometer was obtained by optimising the various parameters. The selective reaction monitoring (SRM) and multiple reaction monitoring (MRM) parameters were set for highest level of sensitivity, as well as extended dynamic range, ensuring superior quantitation performance of MS/MS. The parameters were mentions in Table 1. The production transition positive mode, mass by charge ratio were recorded m/z 130 to m/z 86 for MET by collision induced dissociation (CID) at 112 V and m/z 324 to m/z 110 for GLI by CID at 215 V. The pause time was set 10 ms for better results.

**TABLE 1:** TANDEM MASS SPECTROMETER/SFC WORKING PARAMETERS

MS Parameters		SFC Parameters	
	
Parameter	Value	Parameter	Value
Source temperature MS/MS°	300	Column	Nucleosil C_18_
Nitrogen certain gas (units)	10	Back pressure	20 MPa
Gas 1 (Units)	30	Temperature°	50
Gas 2 (Units)	40	SFC, CO_2_ flow rate ml	2.0
Ion spray voltage, KV	5	SFC, acetonitrile:water (80:20 %v/v), flow rate ml	0.20
Mode of Analysis	Positive	Retention time for MET, min	1.8
Ion transition for MET, m/z	130/86	Retention time for GLI, min	2.3
Collision induced dissociates (CID), V	112	Retention time for I.S, min	2.6
Ion transition for GLI, m/z	324/110		
CID, V	215		
Ion transition for Phenformin	206/105		

#### Preparation of standard and sample solutions:

A standard stock solutions of MET (5 mg/ml) and GLI (1 mg/ml) were prepared by acetonitrile:water (80:20). They were further diluted with same solution when required. A standard stock solution of I.S. was prepared in similar way. Plasma was separated from blood using high speed centrifugation method. The 100 μl plasma was made protein free by treatment with 300 μl of acetonitrile to precipitate proteins. The mixture was then vortex-mixed for 1 min and centrifuged at 10000 rpm for 10 min using polypropylene tube (25 ml). Upper clear layer was collected and 10 μl aliquot of solution was injected into SFC/MS/MS system for analysis.

### RESULTS AND DISCUSSION

**Fig. 2:**
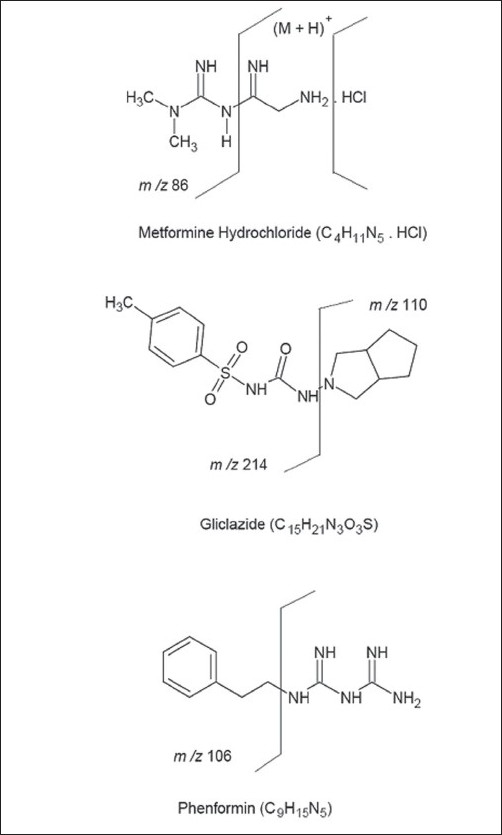
Chemical structures and mass fragmentation pattern Chemical structures and fragmentation pathway of metformin HCl, gliclazide and phenformin.

**Fig. 3:**
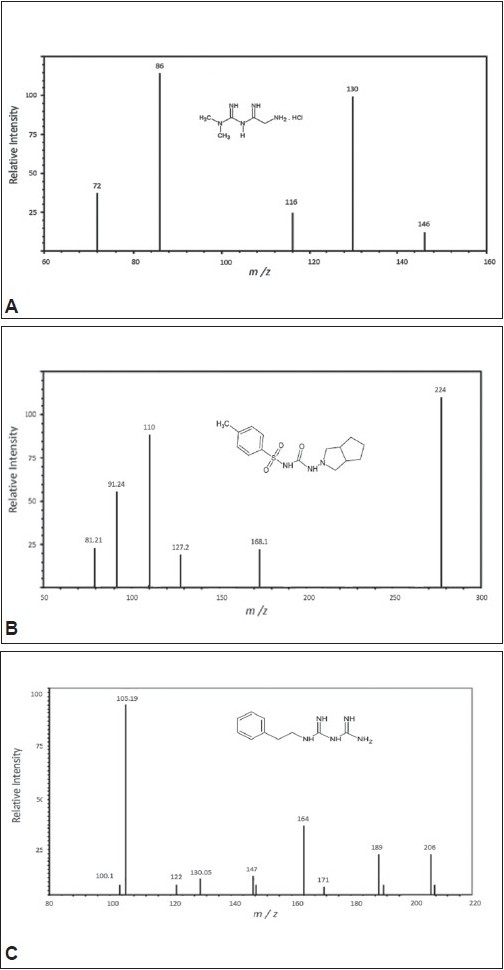
The production mass spectra of [M+H]+ ions. [M+H]+ ions (A) Metformin (MET), (B) gliclazide (GLI) and (C) Phenformin (IS). The spectra were recorded under the identical conditions as SFC in the analysis using liquid CO_2_ mobile phase and acetonitrile:water (80:20).

The chemical structures and proposed fragmentation pathway of MET, GLI and I.S are shown in fig 2. In present study, both positive and negative ionization modes were selected for evaluating the optimum response of MET, GLI and IS. It was found that, the signal intensity of positive ion was higher than the negative ion. Hence the positive ion mode was chosen. The analytes mass spectra were shown in fig. 3. Full scan was done in mass spectra for precursor ion, and found most abundant ion peak [M+H]^+^ at m/z 130 for MET, m/z 324 for GLI and m/z 206 for I.S. The mass by charge ratio in mass spectra was found 130 to 86 for MET and 324 to 110 for GLI and 206 to 105 for I.S. in selected reaction monitoring (SRM) mode for individual product ions. It was observed that the capillary temperature and spray voltage was not influence significantly to the behavior of the compounds in mass spectra. The collision induced dissociation (CID) behavior of the analytes is dependent on the collision energy, which was optimized at 30V, 40V and 50V for MET, GLI and IS, respectively.

Assay selectively was performed by comparing the chromatograms of six different branches of blank human plasma and standard spiked plasma. The plasma samples were collected from healthy human male volunteer after 1 h oral administration of a dispersible tablet containing 500 mg MET and 50 mg GLI. The SRM chromatograms were found 6 ng/ml for MET, 7.5 ng/ml for GLI and 50 ng/ml for I.S. respectively which compared with blank, standard spiked plasma sample in Table 1. There was no significant interference from endogenous substances observed at the retention time of the analytes and I.S. All the ratio of the peak area of the analytes dissolved in the supernatant of processed blank plasma samples compared to the standard solutions at the same concentration levels between 95% and 108%.

**Fig. 4:**
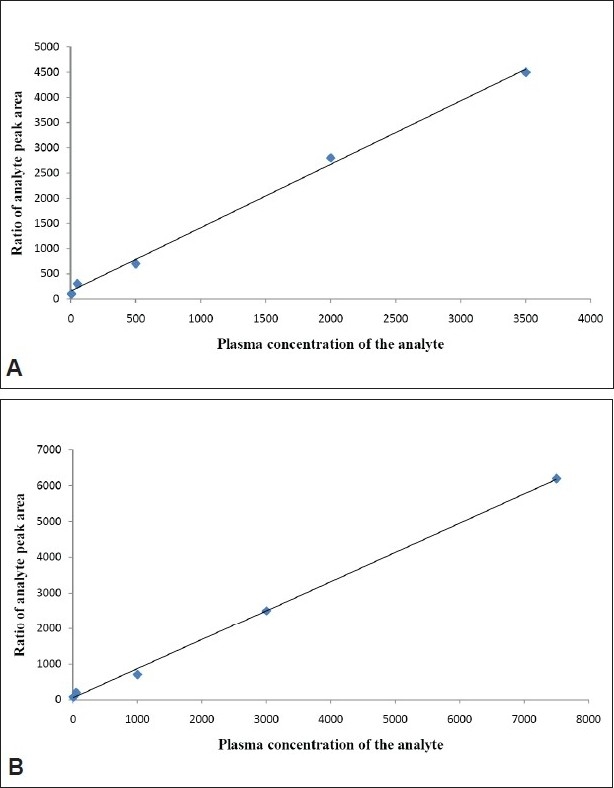
Linear calibration curves of MET and GLI. Linear calibration curves of A. metformin (MET) and B. gliclazide (GLI).

The method was validated by calibration curves, it were constructed based on the measurement of the peak-area ratio of the analytes to I.S. Least squares linear regression analysis was used for carve fitting with 1/x^2^ the weighing factor. Concentration of MET and GLI in the samples were calculated using the equations from appropriate calibration curve fig. 4. The three different concentration levels were analyzed to assess the accuracy and precision of the method. The assays were performed in same day used six replicates of each analytes. Assays were also performed on three separate days at each concentration levels used six replicates. The accuracy was evaluated by the calculation of relative standard deviation (RSD) of the measured concentrations. The intra and inter day precisions were found below 15%. The accuracy was determined by comparing the mean peak areas of the corresponding spike after precipitation of the plasma samples which represented 100% recovery. The spike were prepared after precipitation of samples, blank human plasma was processed according to the sample preparation procedure described above. The upper clean solution layer was collected and then the appropriate standard solutions of MET and GLI were added to give concentrations corresponding to the final concentrations of the pretreated plasma samples. The matrix effects in SFC/MS/MS were determined by comparing the mean peak areas of the spike after precipitation of samples at low, medium and high concentrations (six samples each) with the mean peak areas of the pure samples in equal concentrated.

The stability of the analytes has been studied in plasma at room temperature for 6 h. Extracted validation samples at low, middle and high concentrations were kept at room temperature for over 12 h and were then reanalyzed and quantified against standard curves. Stability of plasma samples were assessed on storage at −50° for 30 days, and the effect of three freeze-thaw cycles were also investigated. The assay was found linear over the concentration range of 6 to 3550 ng/ml for MET and 7.5 to 7000 ng/ml for GLI. All calibration curves fitting with 1/x^2^ weighting scheme. Typical equations of the calibration curves are as follows; MET is y= 1.259x+155.15, r^2^ = 0.9974 and GLI is y= 0.8181x+43.888, R^2^= 0.9985, where y represent the ratio of the analytes peak area to that of IS and x represent the plasma concentration of the analytes. Three each linearity calibration curve and lower limit of quantization was expressed as analytes concentration corresponding to sample blank value plus five standard deviation[14]. The linearity calibration curve and lower limit of quantization was found 6 ng/ml for MET and 7.5 ng/ml for GLI, which were essentials for clinical pharmacokinetic (PK) study.

**Fig. 5:**
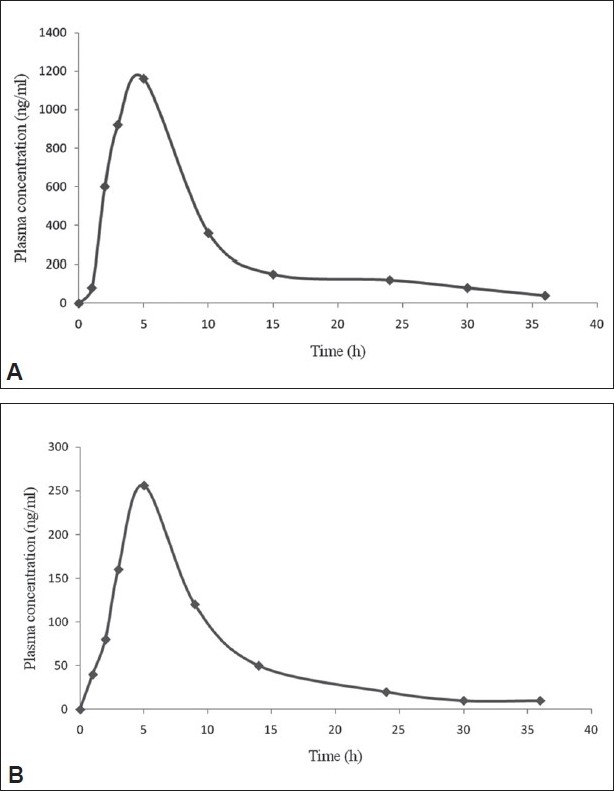
Mean plasma concentration vs. time profile of thirty volunteers Mean plasma concentration vs. time profile of thirty volunteers for A. metformin (MET, 500 mg) and B. gliclazide (GLI, 80 mg) after an oral administration.

Intra-day precision, inter-day precision and accuracy were calculated from data obtained during the ten day validation. Five concentrations were 6, 50, 500, 2000 and 3500 ng/ml for MET and 7.5, 50, 1000, 3000 and 7500 ng/ml for GLI chosen from the high, medium and low range of standard curve fig. 5. Plasma samples spiked at five different concentrations were analyzed at each day of these 10 days validation (n=10 at each concentration). During the 10 days validation the samples were left at room temperature. The values obtained are less than 15% of CV% and RE% in Table 2.

**TABLE 2:** PRECISION AND ACCURACY FOR THE ANALYSIS OF MET AND GLI IN HUMAN PLASMA

Sample	Concentration (ng/ml)		RSD (%)		RE (%)
			
	Spiked	Average obtained	Inter-day	Intra-day	
MET	6.0	6.23	1.05	1.35	0.18
	50	49.06	1.59	1.95	1.15
	2000	2002.08	1.78	1.98	−0.75
GLI	7.5	7.45	1.98	2.01	0.98
	50	49.85	2.05	2.45	−1.15
	1000	1002.58	2.5	2.95	1.45
	3000	3001.95	2.95	3.00	1.35

n=10 days, six replicates per day

**Fig. 6:**
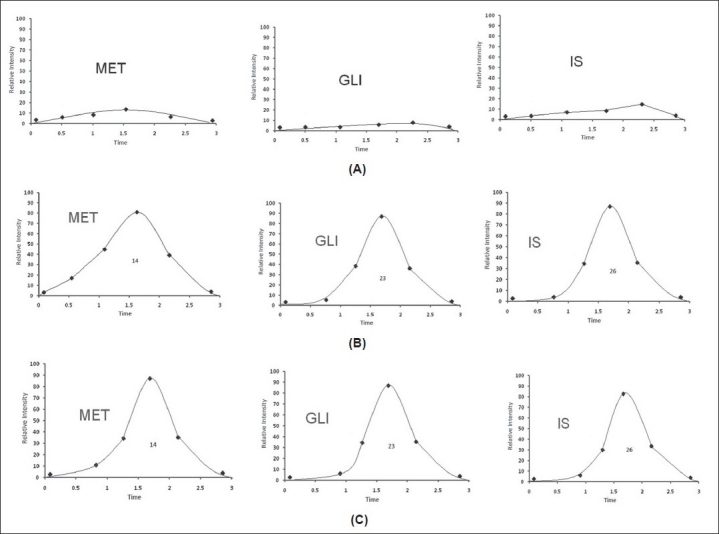
SFC-MRM-Chromatogram for MET, GLI and IS in human plasma samples. SFC-MRM-Chromatogram for metformin (MET), gliclazide (GLI) and phenformin (IS) in human plasma samples; A. blank plasma sample; B. blank plasma sample spiked with MET (6 ng/ml), GLI (7.5 ng/ml) and IS (100 ng/ml); C. volunteer plasma sample 1 h after oral administration of dispersible tablet containing MET (500 mg) and GLI (80 mg)

The mean recovery data of the MET after protein precipitation were found 95, 98 and 102% at the concentration of 6, 100 and 3500 ng/ml while the mean recovery data of GLI were 93, 96 and 93% at the concentration of 7.5, 500 and 7500 ng/ml. The recovery data of the market tablets are given in Table 3. The results of blank plasma samples were gave no response at the retention time mention in fig. 6. The consistency in the method, the assay has proved to be robust in high throughput bio analysis.

**TABLE 3:** ANALYSIS AND RECOVERY OF THE MET AND GLI DISPERSIBLE TABLETS

Brand	Amount of sample ng/ml		Std amount added ng/ml		Recovery ng/ml		% Recovery n = 10	
				
	MET	GLI	MET	GLI	MET	GLI	MET	GLI
I	50	30	-	-	49.53	29.96	-	-
	50	30	10	10	61.05	39.85	101.23	99.58
	50	30	100	50	151.08	80.88	103.05	98.23
	50	30	500	200	548.95	230.58	99.23	101.05
	50	30	1000	1000	1050.99	1002.05	98.23	102.68

To evaluate the possibility of ions suppression or enhancement in the present method, chromatographic peak areas of MET and GLI from the spike after precipitation samples at low, middle and high concentrations levels were compared with these obtained from the standard at the same concentrations. The recovery results obtained at respective concentrations corresponding RSD determined were 93±0.5%, 96±0.7% and 95±0.8% at 10, 50 and 3000 ng/ml for MET and 91±0.7, 96±0.5 and 94±0.9% at 5, 50 and 600 ng/ml for GLI. The resulting chromatographs were also compared with pure samples in same concentrated. It was noticed that there was no significance difference in peak response between these samples. The recovery of the I.S. was found 89±0.9%. Thus, there is no matrix interference in the analytical method of SFC/MS/MS.

The short-term and long-term stability studies were performed on MET and GLI at various temperature and period are given in Table 4. The long term stability studies data of analytes in plasma samples were stored for a period of 30 days at −50°. The stability study showed that the plasma samples of the analytes are stable for three cycles of freeze and thaw within the acceptance criteria of ± 15% of the initial values of the control. MET and GLI plasma samples at −50° and thawed to room temperature. The result of stability studies indicates that, the storage of plasma samples at −50° is adequate, and no stability related problems were found during the routine analysis of samples for pharmacokinetic studies.

**TABLE 4:** STABILITY OF HUMAN SAMPLES OF MET AND GLI

Stability	MET			GLI		
		
	Conc. ng/ml	Mean ng/ml	RSD %	Conc. ng/ml	Mean ng/ml	RSD %
Short term stability for 24 h in plasma	6	5.9	1.52	7.5	7.75	1.10
	100	101	1.15	100	98.93	0.98
	3500	3505	1.65	7500	7505	1.75
Three freeze-thaw cycles	6	6.09	0.95	7.5	7.35	0.98
	100	101.2	1.05	100	101.52	1.25
	3500	3505	1.85	7500	7508	2.05
Auto samples stability 24 h (after extracting and reconstitution)	6	5.97	1.21	7.5	7.45	0.97
	100	102.5	1.65	100	98.75	1.62
	3500	3508	1.89	7500	7503	1.58
Stability for 30 days at ≤ −50°C	6	6.05	0.85	7.5	7.58	1.21
	100	109.2	1.16	100	102.08	1.48
	3500	3509	2.01	7500	7508	1.98

n = 10

The method was validated, the plasma concentration and time profiles are illustrated in Table 5 show the corresponding MET and GLI mean PK parameters. The results showed that the method was simple and selective for the simultaneous determination of MET and GLI in plasma samples.

**TABLE 5:** PHARMACOKINETICS PARAMETERS AFTER ORAL ADMINISTRATION OF COMBINED TABLET TO EACH HEALTHY VOLENTERS

Parameter	MET (500 mg)	GLI (80 mg)
C_max_ (ng/ml)	1156±12	256±8
*T*_max_ (h)	1.56±0.15	1.15±0.08
*T*_1/2_ (h)	3.58±0.30	2.08±0.15
MRT (h)	5.98±0.52	3.96±0.31
AUC_0 - ∞_ (ng * h/ml)	6589±532	958±160
AUC_0 - ∞_ (ng * h/ml)	6723±586	1056±190

It could be concluded that, the SFC/MS/MS method for simultaneous determination of MET and GLI in combined dosage forms is very simple, rapid, robust, sensitive, and hazardous solvent free procedure was used for the first time. The advantages of this method are no sample preparation, no pretreatment and only one step. The method was also successfully applied for the study of PK of the drugs.
